# Foreign

**Published:** 1842-01-22

**Authors:** 


### FOREIGN.

*Influence of the Venereal Disease and sexual gratification upon the union of Fractures.*—The New York Medical Gazette, Vol. II.

page 13, quotes from a paper by M. Thierry, published in L'Experience, Nov. 4, 1841, the following cases:

*Case* 1. M., aged 13, clerk in a wine store, fractured both bones of the fore-arm in descending into the vaults. The apparatus of Boyer was applied, and at the end of sixty days removed, the union being supposed to be perfect. On examination the consolidation was not complete, yet this young man enjoyed good health, and there was nothing to explain why the fracture did not unite. The patient was closely watched, and it was soon discovered that the girl who nursed him afforded him from time to time a physical solace for his ennui; she was discharged, the apparatus reapplied for sixty days, and the consolidation was complete.

*Case* 2. A school-boy of 14 had the arm broken by the fall of a bench. The displacement was inconsiderable, the fracture was reduced and put in the old apparatus; on removing the dressings the bone had not united. The boy was watched, and it was discovered that he was addicted to masturbation. The apparatus was reapplied, and means taken to prevent his recurring to his old habit. The cure was complete.

*Case* 3. M. D., making a false step in the street, fell and fractured the thigh above the great trochanter. The fracture was reduced and the apparatus of Boyer applied. At the end of sixty days, on removing the apparatus, the bone had not united. The patient was asked whether he had not some venereal affection;* on his replying in the affirmative an anti-venereal course was pursued, the apparatus was reapplied, and in twenty-four days the fracture was consolidated.

There is nothing novel in the assertion that the existence of venereal disease retards the union of fractures, though the fact has been frequently disputed; but that sexual indulgence should have the same effect is singular, unless the indulgence be carried to a greater excess than would be probable in those laboring under such injuries. The effects in all the cases are no doubt due to the same cause—the general debility induced by the disease, or the indulgence. That the venereal disease, when not directly involving the periosteum, exerts any specific influence upon a fracture, is extremely improbable;—that it does not always do so is proved by an abundance of testimony, to which, if necessary, we might add some personal observations; but the subject will claim further attention in a future number, when we notice a recent elaborate paper on the occurrence of non-union after fracture, by Dr. Norris.

*M. F. does not tell us whether this was a primary or secondary affection; probably the latter, as he speaks generally of syphilis diminishing the resistance of the bones and preventing their union.

*Origin of Ergot.*—In the Medical Gazette for 1838--9, Edwin Quekett, F.L.S., advanced the doctrine that the formation of Ergot was caused by the introduction of the sporules of a peculiar fungus into the circulation of the affected grain, and their final deposition in the seed, where they found materials and stimuli to induce their developement. In the number of the same journal for October 8, 1841, the following experiments, tending to prove the truth of the doctrine, are narrated.

Twelve healthy grains of rye, of wheat and of barley, (all grown in the neighbouring fields in Surrey,) were selected, and placed in a plate which contained a little water; some ergots of wheat were then immersed in the water of the plate, and with a camel's hair pencil brush the sporidia of the fungus adhering to the exterior were detached in numbers, as the microscope proved, and the ergots were then removed.

A similar experiment was performed with the same number and variety of grains, but with the fungus obtained from the exterior of an ergot of a large grass—*elymus sabulosus:* a glass shade covered each set of grains so prepared.

In a few days germination commenced, and was allowed to progress until the grains were beginning to appear wrinkled, from the appropriation of the albumen; and by this time those that had perfectly germinated possessed green leaves from two to three inches in length. In this state the whole of the young plants were taken into the country, and planted close together in the third week of March last.

The greater number of grains, of both experiments, failed in becoming perfect plants, so that, at the present time, when they are matured, there are but four of rye, (one infested with the fungus from the elymus, and three from wheat,) three of barley, and four of wheat. On every plant of the rye there are some ears possessing ergots (nine having been obtained from the four plants,) some containing one specimen, others as many as six; but in the barley is only one imperfect ergot, and in the wheat not any have been detected.

It was remarked that, in the rye, there was only one ear that possessed a few healthy grains and no ergot; in the others, some had ergots without any healthy grains, and the rest possessed neither ergot nor grains of any kind; showing how the fungus probably influences the formation of healthy grains in this plant, whereas, in the wheat and barley, the sound condition did not appear to have been departed from.

If the cause of the ergots, in this instance, had an external origin, it is singular that, as the plants grow intermixed, and in a very small space, the barley and the wheat should have escaped under the circumstances; but the reason, I imagine, that the latter two possessed no ergots, though treated as the rye, is that they are not so susceptible of the infection; for it is well known that the rye is particularly liable to this disease—more so, perhaps, than any other grass; and that it becomes so, arises either from constitutional properties or its anatomical peculiarities.

I conceive, from these experiments, that the production of ergot from the absorption of the sporules of the previously described fungus, by the fibres of the root of the germinating grains, will be found to be the truc cause of this singular production; and that, when they arrive at the grain, they convert it into the body known as the ergot; for it appears to me too much to admit, in these experiments, that the many ergots on every plant could be the result of accidental circumstances, when it is well known that their presence is very rare in this country on the same grass.

---

*Cases of Dislocation of the Clavicle backwards behind the Sternum.* By M. Morel.—Case I. Lemoine, 17 years of age, was overtaken in a narrow street by a carriage, and had his right shoulder violently squeezed against the wall, in a direction forwards and inwards. He experienced at the time some pain at the bottom of the neck, and a great sensation of suffocation which lasted for more than a quarter of an hour. The dyspnœa gradually subsided, but the motion of the right arm not returning, he, on the eighth day after the accident, entered the *Charité*, when he was found in the following state:—The two shoulders were on the same level, but the right one was nearer the mesial line. The internal extremity of the clavicle was half concealed behind the sternum, and its upper part consequently formed a projection above the bone. When the shoulder was carried backwards, the tumour became more prominent forwards. On depressing the shoulder, it rose, and disengaged itself from behind the sternum, and by carrying the shoulder upwards, outwards, and backwards, the bone returned to its natural position; the hand could be carried to the head, by directing the elbow backwards, but if it was brought forwards, the attempt caused great pain. Desault's bandage was applied; but, as it slipped, it was replaced by that of Velpeau, which kept the bone completely in position. The patient was lost sight of on the 18th day, as he was obliged to be removed to another ward on account of the appearance of small-pox.

Case II.—Lamotte, 28 years of age, was shoeing a horse, when the animal made violent efforts to free his leg; endeavouring to restrain them, he fell, and met with a dislocation of his clavicle backwards. The next day he was in the following state:—The right shoulder was nearer by sixteen lines, than usual, to the mesial line; there was slight swelling of the external jugular vein; some pain was felt, but more towards the middle than the extremity of the clavicle, the external head of which had disappeared behind the sternum, where it was impossible to feel it. When the hand was carried to the head, there was pain if the elbow was moved forwards, but none if it was directed backwards. Two attempts at reduction made, the one by carrying the shoulder outwards and backwards, the other by acting on the arm in the same direction, were unsuccessful.

M. Lenoir made the patient be seated on a low chair; he then placed a sheet round his body, and fastened it to an iron bar. Two assistants pulled the arm and shoulder outwards and backwards, (almost horizontally in fact,) while another kept the wrist lowered, and at the same time turned towards the left side. M. Lenoir then placed his knee between the shoulders, and with one hand pulled the right one backwards, while with the other he traced the clavicle. Reduction was accomplished, and the bone retained in its place by the shoulders being kept back by a bandage applied in the form of the figure 8. Another bandage was placed round the body, and kept the elbow close to the side. On the twelfth day this was removed, and a sling had recourse to. On the eighteenth day he was doing well. The reduction was so complete, that the extremity of the dislocated clavicle was more prominent than that of the other.

The first case put on record of this kind of dislocation, caused by violence, was published by M. Pellieux, in 1834, in the Revue Médicale. Since then, other two cases have been reported in the Gazette Médicale for 1836—'37. Besides the two we have given above, a third is quoted by the Gazette Médicale, from M. Morel's pamphlet; but we have not given it, as it appears to us not to be a case of dislocation backwards. In Sir A. Cooper's Lectures on Surgery, a case is mentioned which was produced by deformity of the spine. The scapula was pushed forward, so that sufficient room was not left for the clavicle between that bone and the sternum, and its internal head gradually slipped behind the latter, pressing upon the œsophagus, and giving rise to great difficulty of swallowing. In this case, as reduction was impossible, the clavicle was sawn through, about an inch from the sternum, and dissected out. The operation was done by Mr. Davis, late of Bungay, in Suffolk. From the cases published, M. Morel says, that there are two kinds of this dislocation, viz. backwards and downwards, and backwards and upwards, as when a part of the inner head of the clavicle is above the level of the sternum.—We can suppose that the latter species is consecutive, and produced by the action of the sterno-mastoid muscle, and by the weight of the superior extremity. The dislocation downwards is much more difficult than the other to reduce, and when reduction is effected, the bones are not so easily retained in their place.—*L. and E. Monthly Journ. Med. Sci.*, Oct. 1841, from *Gazette Medicale*, 21st August 1841.

---

*The Medical Examiner* is published every Saturday by *J. C. Turnpenny*, N. E. corner of Spruce and Tenth streets. Terms—*three dollars* a year for one copy, or *five dollars* for two copies sent to the same post office, in all cases in advance. Postage the same as on newspapers. Letters to be addressed,—post-paid,—to the Editors of the Medical Examiner, Philadelphia.

*Reynell Coates, M. D.*, Acting Editor.
*J. B. Biddle, M. D.*, and *W. W. Gerhard, M. D.*, Co-editors.
*J. B. Biddle, M. D.* Proprietor.

